# Lower functional hippocampal connectivity in healthy adults is jointly associated with higher levels of leptin and insulin resistance

**DOI:** 10.1192/j.eurpsy.2022.21

**Published:** 2022-05-02

**Authors:** Shalaila S. Haas, Alison Myoraku, Kathleen Watson, Thalia Robakis, Sophia Frangou, Fahim Abbasi, Natalie Rasgon

**Affiliations:** 1 Department of Psychiatry, Icahn School of Medicine at Mount Sinai, New York, New York, USA; 2 Department of Psychiatry, Stanford University School of Medicine, Palo Alto, California, USA; 3 Department of Psychiatry, Djavad Mowafaghian Centre for Brain Health, University of British Columbia, Vancouver, British Columbia, Canada; 4 Division of Cardiovascular Medicine, Department of Medicine, Stanford University School of Medicine, Stanford, California, USA

**Keywords:** Connectivity, fMRI, hippocampus, insulin resistance, leptin

## Abstract

**Background:**

Metabolic dysregulation is currently considered a major risk factor for hippocampal pathology. The aim of the present study was to characterize the influence of key metabolic drivers on functional connectivity of the hippocampus in healthy adults.

**Methods:**

Insulin resistance was directly quantified by measuring steady-state plasma glucose (SSPG) concentration during the insulin suppression test and fasting levels of insulin, glucose, leptin, and cortisol, and measurements of body mass index and waist circumference were obtained in a sample of healthy cognitively intact adults (*n* = 104). Resting-state neuroimaging data were also acquired for the quantification of hippocampal functional cohesiveness and integration with the major resting-state networks (RSNs). Data-driven analysis using unsupervised machine learning (*k*-means clustering) was then employed to identify clusters of individuals based on their metabolic and functional connectivity profiles.

**Results:**

*K*-means clustering identified two clusters of increasing metabolic deviance evidenced by cluster differences in the plasma levels of leptin (40.36 (29.97) vs. 27.59 (25.58) μg/L) and the degree of insulin resistance (SSPG concentration: 161.63 (65.27) vs. 125.72 (66.81) mg/dL). Individuals in the cluster with higher metabolic deviance showed lower functional cohesiveness within each hippocampus and lower integration of posterior and anterior components of the left and right hippocampus with the major RSNs. The two clusters did not differ in general intellectual ability or episodic memory.

**Conclusions:**

We identified two clusters of individuals differentiated by abnormalities in insulin resistance, leptin levels, and hippocampal connectivity, with one of the clusters showing greater deviance. These findings support the link between metabolic dysregulation and hippocampal function even in nonclinical samples.

## Introduction

Excess body fact and metabolic dysfunction are epidemic in modern Western societies. Abnormalities in cellular metabolism, influenced by adiposity and glucose dysregulation, affect every tissue in the body, including the central nervous system [1]. Impairments of glucose uptake and insulin signaling in neuronal cells have been associated with deficits in neuronal plasticity and cognitive function [2]. However, a detailed understanding of the effects excess adiposity and metabolic impairment may have on higher-order cognitive function in humans remains elusive.

Resting-state functional magnetic resonance imaging (rs-fMRI) has been fundamental in uncovering the functional architecture of the brain as inferred from the spontaneous fluctuations in the blood-oxygen-level-dependent (BOLD) signal. Resting-state connectivity, defined as correlations between these spontaneous BOLD fluctuations, has been used to define the spatiotemporal characteristics of resting-state networks (RSNs) [3, 4]. The major and most consistent RSNs can be divided into those supporting internally guided, higher-order mental functions (i.e., the default-mode, central executive, and salience networks) and those supporting externally driven, specialized sensory and motor processing (i.e., visual and sensorimotor networks) [5–8].

The default mode network (DMN) is considered the backbone of information integration in the brain [9, 10]. A major subcortical hub of the DMN is the hippocampus, a medial temporal lobe structure, that contributes to a wide array of cognitive functions, notably episodic memory [11], visuospatial navigation [12], detection of contextual deviation [13, 14], and emotional reactivity [15]. A rich literature, informed by cross species studies, has provided a detailed description of the basic hippocampal anatomy and circuitry. The hippocampus is divided into the dentate gyrus (DG), the cornu ammonis (CA) fields, and the subiculum, based on their distinctive cytoarchitectural and transcriptional profiles [16, 17]. This “trisynaptic pathway” is functionally organized into gradient domains along its longitudinal axis; information generally enters into the DG through the perforant pathway and flows through the CA fields to reach CA1 and the subiculum, which project to multiple brain regions [18–21].

Impaired hippocampal integrity is common in numerous and diverse neuropsychiatric disorders with the DG and CA1 being the most consistently implicated hippocampal subfields [22–27]. It has been proposed that the mechanisms underlying hippocampal pathology across disorders may involve abnormalities in the biological pathways subserving energy homeostasis and stress response. In preclinical models, insulin resistance, defined as reduced responsiveness of the insulin-signaling pathways, has been shown to impair the structural and functional integrity of the hippocampus through its adverse impact on dendritic spine and synapse formation, activity-dependent synaptic plasticity, and neurogenesis [28–30]. In humans, insulin resistance is the hallmark of type 2 diabetes mellitus and has been associated with hippocampal hypoconnectivity [31–33] that can be reversed following administration of intranasal insulin [34]. Adiposity, typically expressed as body mass index (BMI) and waist circumference (WC), is a further risk factor for hippocampal dysfunction, acting through multiple pathways which critically involve leptin, a hormone secreted by adipocytes [35]. Adiposity is also closely linked to over-activation of the hypothalamic–pituitary–adrenal (HPA) axis [36] which may contribute to hippocampal dysfunction because of the high concentration of cortisol receptors in the hippocampus [37]. Cortisol, a stress hormone, can be elevated in obese individuals as an inflammatory response and as an allostatic stress factor [38]. Therefore, measures of adiposity, insulin resistance, and HPA activation show a tight functional association that influences the metabolic status of each individual.

The aim of the current study was to expand current understanding of the effect of insulin resistance, adiposity, and HPA function on the resting-state connectivity of the hippocampus in healthy and cognitively intact adults. We focused on the internal functional cohesiveness of the left and right hippocampus and their functional integration within the wider intrinsic architecture of the brain. Furthermore, aligned with the notion that hippocampal functional integrity is jointly defined by the circulating levels of insulin, leptin, and cortisol, we used unsupervised clustering to test the hypothesis that healthy individuals can be stratified into subgroups based on their metabolic and hippocampal functional connectivity profiles.

## Methods

### Sample recruitment

One hundred and twenty-six adult men and women were recruited by advertisement from the campus and surrounding neighborhoods of Stanford University. Enrolled participants were screened to exclude individuals with endocrine, neurological, cardiovascular, and psychiatric disorders or contraindications to magnetic resonance imaging (details in the Supplementary Material). Ethical approval was obtained from the Human Subjects Committee of Stanford University, and all participants provided written informed consent.

### Procedures

The study involved two visits within a month, at the Stanford Clinical and Translational Research Unit and the Center for Cognitive and Neurobiological Imaging.

#### Cognitive evaluation

An estimate of current intelligence quotient was assessed using the Wechsler Abbreviated Scale of Intelligence, Second Edition [39], and two of its subtests, the Digit Symbol-Pairing and Digit-Symbol-Free Recall, were used to evaluate episodic memory.

#### Anthropometric measures of fat mass

Participants’ WC, height (in meters), and weight (in kilograms) were determined using standardized procedures (details in the Supplementary Material). The BMI was then calculated as weight/height squared.

#### Laboratory assessments

After a 10-h overnight fast, blood samples were drawn for measurement of fasting plasma insulin (FPI), fasting plasma glucose (FPG), leptin levels, and cortisol levels (details in the Supplementary Material).

#### Modified insulin suppression test

Immediately following the blood draw for the laboratory tests, participants underwent the insulin suppression test (IST) [40, 41] for the quantification of insulin-mediated glucose uptake (details in the Supplementary Material). The IST is an established test comparable to the more invasive and laborious euglycemic clamp [42]. During the IST, the endogenous insulin secretion is suppressed by octreotide acetate infusion, and hepatic glucose production is suppressed by the combination of physiological hyperinsulinemia and glucose infusion. The steady-state plasma insulin concentrations achieved in this fashion are similar across individuals, and the magnitude of the steady-state plasma glucose (SSPG) provides a direct measure of peripheral insulin sensitivity; higher SSPG concentration reflects greater insulin resistance.

#### Neuroimaging acquisition and preprocessing

Anatomical T_1_ images and rs-fMRI data were acquired in all participants using a 3T GE Discovery MR750 scanner (https://www.gehealthcare.com/products/magnetic-resonance-imaging/3-0t/discovery-mr750). All preprocessing was carried out using Statistical Parametric Mapping software (SPM12; https://www.fil.ion.ucl.ac.uk/spm/software/spm12/). Details of the acquisition sequences, quality assurance, and data processing are provided in the Supplementary Material.

#### Functional connectivity

In each participant, the Anatomy Toolbox [43] in SPM12 was used to define maximum probability regions of interest (ROIs) in Montreal Neurological Institute (MNI) space corresponding to the whole hippocampus, the DG, and the CA1/subiculum in each hemisphere. In addition, the central executive network (CEN), salience network (SAL), visual network (VIS), and sensorimotor network (SMN) were defined using templates provided by the consensual atlas of resting-state network (CAREN) [44] (details in the Supplementary Material and Supplementary Figure S1).

Hippocampal cohesiveness (i.e., functional connectivity within the hippocampus) was computed separately for the left and right hippocampus; in each hippocampus, cohesiveness was calculated as the Fisher *Z*-transformed Pearson’s correlation coefficient of the average BOLD signal time series of each hippocampal voxel with that of all the other hippocampal voxels. Prior studies have shown that the anterior and posterior hippocampus have different connectivity profiles [18–21]. In the human neuroimaging literature, the uncal apex has been typically used to define the anterior and posterior segments; however, this approach results in segments with a mixture of hippocampal subfields and does not accommodate inter-individual variability [19]. Here, we used the ROIs defined in the left and right DG and CA1/subiculum as, respectively, representative of the posterior and anterior segments of the left and right hippocampus. The functional integration of the hippocampus within the intrinsic functional architecture of the brain was computed as the correlation between the average BOLD signal time series of each of the CAREN-defined RSNs and that of each posterior and anterior segment.

### Statistical analyses

Out of 126 participants, only 104 had undergone both neuroimaging and direct measures of insulin resistance during the IST. Descriptive statistics were used to summarize the sample characteristics. After regressing-out the effect of age and sex, the Spearman’s correlation coefficient was used to assess the association matrix of the metabolic and the functional connectivity measures. As these associations represent the basic matrix of the dataset, statistical significance was reported at *p* < 0.05.

Participants were classified into clusters according to proximity criteria by means of the *k*-means algorithm implemented in *R* using version 3.0 of the NbClust package (https://cran.r-project.org/web/packages/NbClust/NbClust.pdf) using the BMI, WC, SSPG, FPI, FPG, leptin levels, and cortisol levels, and the hippocampal cohesiveness and integration measures as input data. We included both metabolic and connectivity measures as input features into the *k*-means algorithm as we considered them as a compound phenotype emerging through complex interactions between the individual features. An alternate approach which considers only the metabolic blood-based measures as input features is presented in the Supplementary Material.

In the algorithm, *K* points, serving as initial cluster centroids, were placed into the space represented by the sample. Then each participant was assigned to a cluster based on its proximity to its centroid. When all participants had been assigned, the positions of the *K* centroids were recalculated. The process is then repeated until the centroids remain unchanged. This process separates participants into homogenous clusters while maximizing heterogeneity between clusters. The number of clusters was identified based on the solution endorsed by majority vote of fit indices. The clusters of the optimal solution were compared in terms of sex, ethnicity, and average and maximum volume-to-volume head displacement.

## Results

The sample of the analyses comprised 104 individuals for whom complete data were available in Supplementary Table S1. The majority of participants (*n* = 89; 86%) had normal FPG levels (<100 mg/dL), whereas in the remainder (*n* = 15; 14%) could be considered prediabetic as their FPG levels ranged between 100 and 120 mg/dL [45]. Furthermore, 14 (13%) participants had a normal BMI, whereas 53 (51%) were overweight and 37 (36%) were obese. Sex- and age-adjusted correlations among all the measures examined are provided in Table 1 and Supplementary Tables S2 and S3. With the exception of the cortisol levels, all metabolic measures were significantly correlated with one another (all *p* < 0.001; Supplementary Table S2).

**Table 1.** Age- and sex-adjusted Spearman correlation coefficients (Rho) among measures of fat mass, insulin-dependent glucose uptake, leptin, and cortisol and hippocampal functional connectivity measures with the resting-state networks.

		SSPG	FPI	FPG	BMI	WC	Leptin	Cortisol
*Left*								
CA1/Sub-CEN	Rho-value	0.21	0.15	0.04	0.14	0.21	0.18	−0.26
	*p*-value	0.04	0.13	0.67	0.17	0.03	0.07	0.01
CA1/Sub-SAL	Rho-value	−0.19	−0.16	−0.05	−0.16	−0.17	−0.20	−0.03
	*p*-value	0.05	0.12	0.61	0.11	0.08	0.04	0.75
CA1/Sub-SMN	Rho-value	−0.23	−0.17	−0.10	−0.24	−0.27	−0.19	0.06
	*p*-value	0.02	0.09	0.30	0.01	0.005	0.05	0.53
CA1/Sub-VIS	Rho-value	−0.07	−0.11	0.11	−0.005	−0.05	−0.05	0.05
	*p*-value	0.49	0.28	0.29	0.96	0.61	0.59	0.59
DG-CEN	Rho-value	0.09	0.15	−0.03	0.21	0.20	0.21	−0.19
	*p*-value	0.37	0.14	0.80	0.04	0.04	0.03	0.05
DG-SAL	Rho-value	−0.23	−0.16	−0.05	−0.05	−0.16	−0.13	0.003
	*p*-value	0.02	0.11	0.61	0.59	0.11	0.19	0.98
DG-SMN	Rho-value	−0.16	−0.10	−0.06	−0.09	−0.18	−0.10	0.18
	*p*-value	0.10	0.31	0.54	0.37	0.06	0.34	0.07
DG-VIS	Rho-value	0.004	−0.04	0.23	0.11	0.07	0.09	0.06
	*p*-value	0.97	0.67	0.02	0.26	0.47	0.34	0.55
*Right*								
CA1/Sub-CEN	Rho-value	0.12	0.21	0.10	0.17	0.20	0.11	−0.07
	*p*-value	0.23	0.04	0.31	0.09	0.04	0.28	0.50
CA1/Sub-SAL	Rho-value	−0.14	−0.14	−0.02	−0.02	−0.13	−0.17	0.002
	*p*-value	0.14	0.16	0.82	0.88	0.20	0.08	0.98
CA1/Sub-SMN	Rho-value	−0.28	−0.22	−0.12	−0.19	−0.32	−0.25	0.09
	*p*-value	0.004	0.02	0.22	0.05	0.001	0.01	0.35
CA1/Sub-VIS	Rho-value	−0.03	−0.03	0.12	−0.02	−0.09	−0.06	0.08
	*p*-value	0.72	0.73	0.24	0.87	0.38	0.53	0.42
DG-CEN	Rho-value	0.04	0.13	0.09	0.06	0.11	0.13	−0.20
	*p*-value	0.71	0.18	0.35	0.52	0.25	0.19	0.04
DG-SAL	Rho-value	−0.22	−0.13	−0.08	−0.07	−0.16	−0.10	0.01
	*p*-value	0.03	0.19	0.42	0.45	0.10	0.31	0.90
DG-SMN	Rho-value	−0.15	−0.16	−0.06	−0.11	−0.23	−0.10	0.14
	*p*-value	0.13	0.11	0.53	0.25	0.02	0.33	0.17
DG-VIS	Rho-value	0.002	−0.08	0.13	−0.03	−0.11	−0.09	0.06
	*p*-value	0.98	0.40	0.18	0.74	0.27	0.37	0.58

Abbreviations: BMI, body mass index; CA1, cornu ammonis 1; CEN, central executive network; DG, dentate gyrus; FPI, fasting plasma insulin, FPG, fasting plasma glucose; SAL, salience network; SMN, sensorimotor network; SSPG, steady-state-plasma glucose; Sub, subiculum; VIS, visual network; WC, waist circumference.

**Figure 1.**
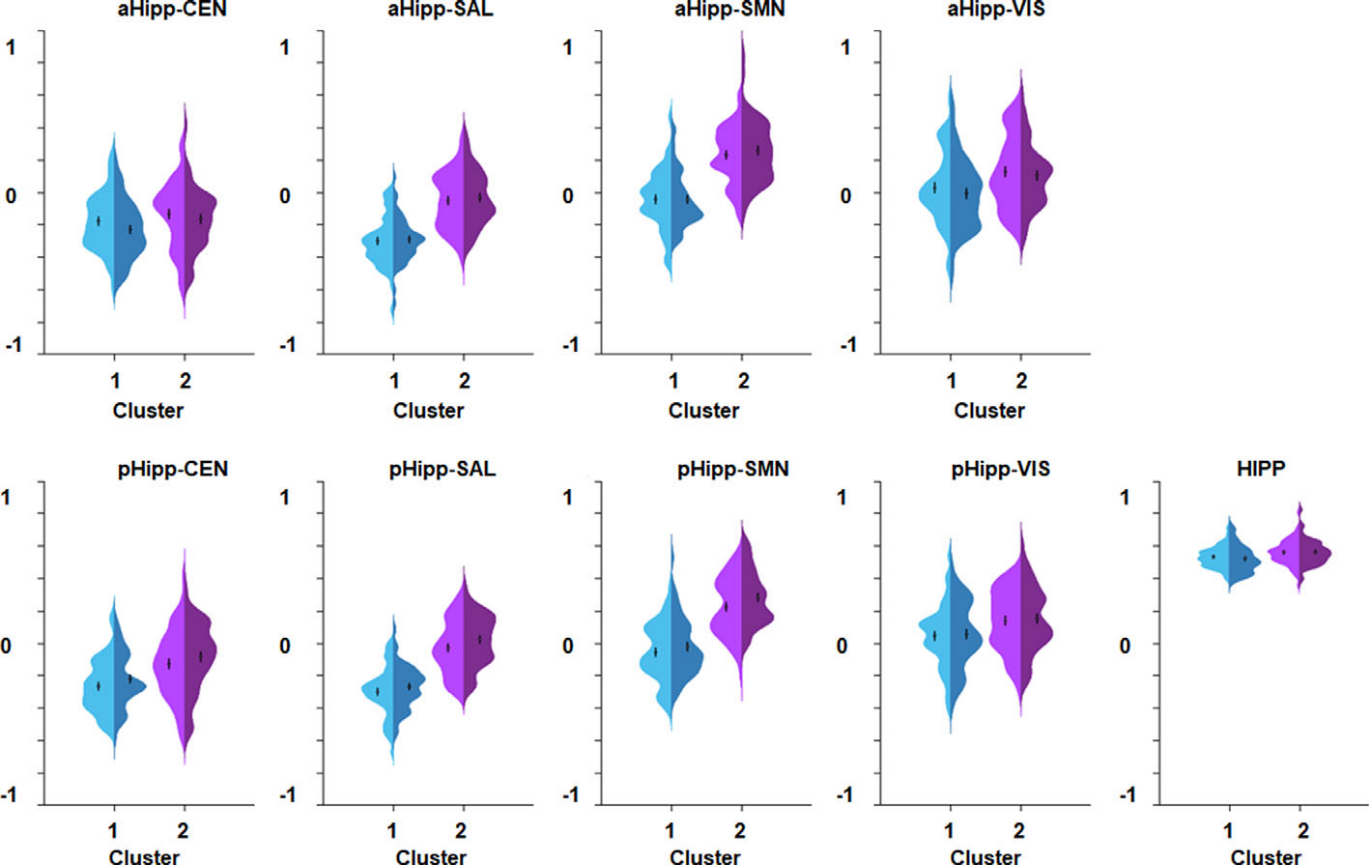
Cluster differences in hippocampal functional cohesiveness and integration. Violin plots of the distribution and differences in the hippocampal functional connectivity in Clusters 1 and 2; Cluster 1 showed significantly lower hippocampal cohesiveness (i.e., within-hippocampus connectivity) and integration (i.e., connectivity between the left and right anterior and posterior hippocampal segments with each RSN). Cluster 1 is depicted in blue and Cluster 2 in purple; within each cluster, the lighter hue corresponds to the left side and the darker hue to the right side. All measures of connectivity depicted are statistically significantly different between the clusters. The box plots represent the mean and standard error. *Abbreviations:* aHipp, anterior hippocampal segment; CEN, central executive network; Hipp, hippocampal cohesiveness; pHipp, posterior hippocampal segment; SAL, salience network; SMN, sensorimotor network; VIS, visual network.

*K*-mean clustering identified two clusters with a majority vote of seven fit indices (whereas other solutions were supported by three or less). Individuals in Cluster 1 (*n* = 51) had higher SSPG and leptin levels and lower hippocampal cohesion and integration compared to those in Cluster 2 (*n* = 53; Table 2 and Figure 1). Individuals in Cluster 1 were more likely to be female (*p* = 0.002), but did not differ in cognitive or any other sociodemographic feature (Table 2). In addition, there were no significant cluster differences in either the average or the maximum head displacement (*p* > 0.05). These findings were recapitulated in supplemental analyses in which only metabolic measures were used as input features for clustering (details in the Supplementary Material and Supplementary Figure S2).

**Table 2.** Sociodemographic and metabolic characteristics of the clusters.

Variable	Cluster 1 (*N* = 51)	Cluster 2 (*N* = 53)	*p*-Value
*Sociodemographic measures*			
Sex, female, *N* (%)	40 (78%)	26 (49%)	0.002
Age, years, mean (SD)	40.04 (7.89)	38.23 (8.98)	0.28
Ethnicity			0.50
White, *N* (%)	19 (49%)	37 (57%)	
Asian, *N* (%)	12 (31%)	20 (31%)	
Other, *N* (%)	8 (20%)	8(12%)	
Education, years, mean (SD)	17.66 (5.09)	17.25 (3.38)	0.63
*Cognitive measures*			
IQ, mean (SD)^a^	117.55 (10.02)	120.68 (13.62)	0.19
Digit symbol pairing, mean (SD)^a^	13.55 (4.86)	13.92 (4.90)	0.70
Digit symbol free recall, mean (SD)^a^	7.90 (1.14)	7.96 (1.07)	0.78
Mini mental state examination, mean (SD)	28.71 (1.27)	28.89 (1.24)	0.46
*Anthropometric measures of fat mass*			
WC, m, mean (SD)^b^	0.98 (0.12)	0.97 (0.09)	0.41
BMI, kg/m^2^, mean (SD)^b^	29.6 (4.5)	28.1 (3.4)	0.48
Normoweight, *N* (%)	10 (20%)	9 (17%)	0.36
Overweight, *N* (%)	20 (39%)	28 (53%)	
Obese, *N* (%)	21 (41%)	16 (30%)	
*Laboratory measures*			
Fasting leptin, μg/L, mean (SD)^b^	40.36 (29.97)	27.59 (25.58)	0.007
Fasting glucose, mg/dL, mean (SD)^b^	91 (10)	89 (10)	0.39
SSPG, mg/dL, mean (SD)^b^	161 (65)	126 (67)	0.005
Fasting insulin, mU/L, mean (SD)^b^	10.60 (7.34)	9.46 (4.59)	0.50
Cortisol, μg/L, mean (SD)^b^	8.69 (4.58)	9.19 (4.87)	0.68

Note: Normoweight: 21 ≤ BMI ≤ 25; Overweight: 26 ≤ BMI <29; Obese: 30 ≤ BMI ≤ 41.

Abbreviations: BMI, body mass index; IQ, intelligence quotient; SD, standard deviation; SSPG, steady-state plasma glucose; WC, waist circumference.

a Cognitive measures were assessed using the Wechsler Abbreviated Scale of Intelligence, Second Edition.

b Mann–Whitney *U* tests employed for variables with nonnormal distribution.

## Discussion

In this study, we aimed to assess hippocampal functional connectivity in relation to the anthropometric and peripheral metabolic measures. We identified two distinct clusters that differed in hippocampal functional connectivity and metabolic function. Relative to Cluster 2, Cluster 1 comprised individuals with higher leptin, a greater magnitude of insulin resistance, lower hippocampal cohesion and integration, but intact selective cognitive domains of interest.

The current study further expands our earlier investigation of 20 healthy women at risk for Alzheimer’s disease (AD) in whom higher insulin levels were associated with lower hippocampal-prefrontal resting-state connectivity [46]. Other groups have reported hippocampal “hypoconnectivity” at rest, albeit with diverse inter-study patterns, in elderly individuals with episodic memory difficulties [23, 47] as well as in individuals with mild cognitive impairment or incipient dementia [48]. The present findings that higher levels of leptin and greater insulin resistance cluster together with lower hippocampal functional cohesiveness and integration suggest that hippocampal “hypoconnectivity” may represent an early sign of hippocampal distress that predates cognitive dysfunction. This notion is also consistent with findings in AD where hippocampal functional deterioration predates cognitive decline [49]. Although firm conclusions must await longitudinal studies, the present observations strongly indicate that lower hippocampal connectivity may be an informative biomarker of diminished hippocampal integrity in metabolically challenged but otherwise healthy individuals.

Investigations of the association between metabolic measures and neuroimaging variables have typically relied on regression analyses which assume that the predictor variables represent independent observations with uniform linear associations across the entire sample. However, as we have shown here, measures of insulin-dependent-glucose uptake and adiposity are highly correlated (Supplementary Table S2) and prior literature has shown that their association is complex and interactive: obesity engenders insulin resistance [50, 51], but not all overweight or obese individuals are insulin resistant [52]. Therefore, the internal milieu of each individual person is likely to reflect the joint influences of these metabolic pathways.

Accordingly, we used a person-based data-driven approach to identify clusters of individuals based on their combined metabolic and hippocampal connectivity characteristics. We identified two clusters of increasing metabolic deviance primarily differentiated by the plasma levels of leptin and the degree of insulin resistance, as inferred by the SSPG. Leptin is an adipocytokine, secreted primarily from white adipose tissue, whose circulating levels correlate directly with body fat mass [53]. Women generally have higher leptin levels than men which may explain their overrepresentation in Cluster 1 [54]. Leptin enters the brain readily and regulates satiety and food intake through its action on hypothalamic circuits [55]. Leptin and insulin are linked through a bidirectional feedback loop, referred to as the adipoinsular axis [56], whereby leptin inhibits insulin synthesis and secretion, whereas insulin stimulates leptin secretion.

The hippocampus is rich in both insulin and leptin receptors [57, 58]. Preclinical studies have shown that insulin-mediated glucose uptake is critical for hippocampal dendritic spine and synapse formation [59], activity-dependent plasticity, and neurogenesis [60]. However, the effect of leptin on the hippocampus is more complex; adaptive leptin-induced regulation of hippocampal synaptic plasticity by leptin occurs within a narrow concentration range, so that both low and high leptin levels are ineffectual [61].

Resting-state functional connectivity and data-driven clustering are powerful tools for examining the association between hippocampal connectivity and the metabolic status. Our methods are further strengthened by rigorous neuroimaging quality control and elimination of images potentially compromised by motion artifacts; nevertheless, the present results derive from a single sample, and thus replication is critical. We chose RSN templates from CAREN [44] because this approach enhances replicability but acknowledge that there are other ways to define RSNs. We defined the anterior and posterior portions of the hippocampus using probabilistic maps which are more reliable and informative than landmark-based divisions that use the uncal apex; however, the micro-level complexity of the hippocampal circuitry cannot be fully accessed using macro-level neuroimaging measures. The connectivity analyses were correlational and cross-sectional, and therefore they cannot establish causative links with the metabolic variables.

In conclusion, we identified two clusters of individuals differentiated by the degree of abnormalities in insulin resistance, leptin levels, and hippocampal hypoconnectivity. These findings demonstrate the link between metabolic dysregulation and hippocampal function even in nonclinical sample. Furthermore, planned follow-up assessment of the current sample will enable examination of the longitudinal trajectories of the association between metabolic risk and hippocampal function.

## Data Availability

Data that support these findings are available upon request from the PI (N.R.) of the study.

## Supplementary material

For supplementary material accompanying this paper visit https://doi.org/10.1192/j.eurpsy.2022.21. click here to view supplementary material
