# Supplementary material for: Lower functional hippocampal connectivity in healthy adults is jointly associated with higher levels of leptin and insulin resistance
Source: Eur Psychiatry. 2022 May 2;65(1):e29. doi: 10.1192/j.eurpsy.2022.21 (PMC9158395; doi:10.1192/j.eurpsy.2022.21)
Supplement: Supplementary file 1 [file S0924933822000219sup001.docx]

**Lower functional hippocampal connectivity in healthy adults is jointly associated with higher levels of leptin and insulin resistance**

**Supplementary Material**

1. **Supplementary Methods**

**A.1. Sample recruitment**

Adult men and women were recruited via flyers distributed on the campus of Stanford University and in the surrounding communities. Potential participants were screened for the following inclusion criteria: (i) at least 12 years of education, (ii) cognitively intact as defined by a score of > 27 of the Mini Mental Status Exam [1]. Participants were excluded for any of the following: (i) diagnosis of possible or probable dementia or mild cognitive impairment; (ii) history of Type I or II diabetes; (iii) significant cardiovascular disease including unstable or untreated hypertension, myocardial infarction or cerebrovascular events; (iv) significant medical or neurological conditions including pulmonary disease, endocrine disorders and cancer; (v) history of head trauma; (vi) use of any medications that may significantly affect the insulin uptake (e.g., systemic corticosteroids, anti-diabetics, or anti-cholesterol medications); (vii) any lifetime psychiatric disorder and alcohol/ substance abuse or dependence within the past six months based on the structured clinical interview for the DSM-IV axis I disorders [2]; (viii) any past psychiatric illness; (ix) current clinical depression define as a score of < 8 of the 17-item Hamilton Depression Rating Scale; (x) contra-indications to magnetic resonance imaging (MRI) (e.g., metallic object in the body, claustrophobia). The characteristics of the enrolled sample are shown in Table 1.

**A.2. Anthropometric Measurements**

Height and weight were measured while participants were wearing light clothing and no shoes. Waist circumference was measured at the midpoint between lower rib cage and upper iliac crest in mid respiration while the participants were standing.

**A.3. Modified Insulin Suppression Test**

After a 10-hour overnight fast, an intravenous catheter was placed in the antecubital vein of each arm; one arm vein was used for drawing blood samples and the other for giving a simultaneous, continuous infusion of octreotide acetate (0.27 μg/m2/min), insulin (32 mU/ m2/min), and glucose (267 mg/m2/min) for 180 min. Blood samples were drawn every 30 min until 150 min to monitor plasma glucose concentrations and then every 10 min until 180 min to measure plasma glucose and insulin concentrations. The plasma glucose and insulin concentration values during the last 30 min of the infusion were averaged to determine the steady-state plasma glucose (SSPG) and steady-state plasma insulin (SSPI) concentrations, respectively.

**A.4 Laboratory Measurements**

Blood was drawn after a 10-hour overnight fast. Plasma glucose was measured at the Stanford Clinical and Translational Research Unit on a Nova Glucose Analyzer (Nova Biomedical Waltham, MA, USA). Plasma insulin, leptin and cortisol were measured at the Core Laboratory for Clinical Studies at Washington University School of Medicine, USA. Insulin was measured by radioimmunoassay (Millipore, St. Charles, MO). The intra and inter-assay coefficient of variation for insulin ranged between 5.1 to 7.0% and 6.4 to 8.4%, respectively.

**A.5 Neuroimaging**

All neuroimaging data were acquired at a single visit on a 3T system (Discovery MR750w; GE Healthcare, Milwaukee, Wisconsin) located at the Center for Cognitive and Neurobiological Imaging, Stanford University.

*A.5.1 Structural MRI Acquisition*: Whole-brain T1-weighted images in all participants were acquired using a three-dimensional inversion recovery prepared fast spoiled gradient recalled acquisition (3D IR FSPGR) with the following parameters: voxel size 1 mm isometric, Repetition Time (TR): minimum, Time to Echo (TE): minimum, flip: 12 degrees, Inversion Time (TI): 300 ms, field of view (FOV): 200x200 mm^2^, 190 slices, slice thickness: 0.9 cm, 256x256, and NEX:1.

*A.5.2 Resting-state fMRI Acquisition*: rs-fMRI data were acquired using the following parameters:

TR: 2000 ms, TE: 30 ms, flip: 77 degrees, FOV: 200 x 200 mm^2^, 32 slices, slice thickness: 3mm, matrix: 256x256, and in-plane spatial resolution: 2.9 mm.

*A.5.3. Quality Assurance*

Following a protocol developed for the Human Connectome Project [3] raw T_1_ images were inspected for crispness, blurriness, motion and other artifacts and were rated on 4 point scale (1 = poor, 2 = fair, 3 = good, 4 = excellent). All images were rated as fair or better.

To minimise motion-related artefacts, scans were included if maximal volume-to-volume head displacement was <3 mm translation or 0.5° rotation [4]. Four individuals were excluded on based on these criteria; the remainder had average mean displacement was 0.12 (none with average mean displacement >0.5) and average maximal displacement was <0.6.

*A.5.4 Structural MRI Preprocessing:* Structural imaging data were analyzed using the Statistical Parametric Mapping (SPM) 12 Toolbox (https://www.fil.ion.ucl.ac.uk/spm/software/spm12/) version 7487 to define hippocampal subfields using the segmentation algorithm available in in the SPM Anatomy Toolbox. Probabilistic regions of interest (ROIs) were defined in each participant corresponding to the whole hippocampus, the dendate gyrus (DG) and the cornu ammonis 1 (CA1)/subiculum, in the left and right hemisphere.

*A.5.5 Resting-state fMRI Preprocessing:* All rs-fMRI data were preprocessed using the Statistical Parametric Mapping (SPM) 12 Toolbox (https://www.fil.ion.ucl.ac.uk/spm/software/spm12/) version 7487. Preprocessing included the following steps: discarding of the first 3 volumes, motion correction to the first volume with rigid-body alignment; co-registration between the functional scans and the anatomical T_1_ scan; spatial normalization of the functional images into MNI stereotaxic standard space; spatial smoothing with a 6-mm at full-width at half-maximum Gaussian kernel. Data were additionally preprocessed to correct for head motion using detrending and multiple regression of motion parameters and their derivatives (24-parameter model) [5], as well as of white matter (WM) and cerebrospinal fluid (CSF) time series and their linear trends. Lastly, a bandpass filter (0.01-0.08Hz) was applied [6]. The time course of head motion was obtained by estimating the translations in each direction and the rotations in angular motion about each axis for each volume.

*A.5.6 Definition of the Major Resting-State Networks*

The Consensual Atlas of REsting‐state Network (CAREN) was used to extract the major resting-state network (RSNs) networks. CAREN was developed to address inter-study uncertainty with regards to the spatial definition of RSNs based on their optimal consensual definition across available brain functional atlases derived from large samples (>100) of healthy individuals. It is the RSN templates provided by CAREN are independent of neuroimaging parameters (site, acquisition sequence and analytical methods) and sample composition. The network templates can be freely accessed at https://www.researchgate.net/publication/334042115_Consensual_Atlas_of_REsting-state_Networks_CAREN and are shown in Supplementary Figure S1.

**A.6 K-means Clustering**

The documentation for the K-mean clustering used here is free available at https://www.rdocumentation.org/packages/NbClust/versions/3.0/topics/NbClust. The package provides 30 indices for determining the number of clusters and derives the best clustering scheme from the different results obtained by varying all combinations of number of clusters, distance measures, and clustering methods.

Two approaches were used with regards to input features: (a) in the main manuscript metabolic measures (i.e., BMI, WC, SSPG, FPI, FPG, leptin levels, and cortisol levels) and the hippocampal cohesiveness and integration measures were all entered as input features; (b) in the supplement we used only the metabolic measures as input features and then resultant clusters were then compared in terms of their hippocampal functional connectivity measures.

| **Supplementary Table S1. Characteristics of the analysis sample (N=104)** | | |
| --- | --- | --- |
| **Sociodemographic Measures** | |  |
| Age, years [Mean (SD)] | 39.12 (8.47) |  |
| Female Sex, N(%) | 66 (63.46%) |  |
| Ethnicity -White, N (%)  Ethnicity- Asian, N (%)  Ethnicity-Other, N (%) | 56 (53.9%)  32 (30.8%)  16 (15.4%) |  |
| Education, years [Mean (SD)] | 17.45 (4.29) |  |
| **Anthropometric Measures of Fat Mass** | |  |
| Body Mass Index, kg/m^2^ [Mean (SD)] | 29.21 (3.96) |  |
| Body Mass Index, range | 21.2-40.5 |  |
| Waist circumference, m [Mean (SD)] | 0.97 (0.11) |  |
| **Laboratory Measures** | | |
| Fasting glucose, mg/dL [Mean (SD)] | 90.27 (9.78) |  |
| SSPG, mg/dL [Mean (SD)] | 143.33 (68.16) |  |
| Fasting Insulin, mU/L [Mean (SD)] | 10.02 (6.09) |  |
| Fasting Leptin, µg/L [Mean (SD)] | 33.85 (28.42) |  |
| Cortisol, µg/L [Mean (SD)] | 8.95 (4.72) |  |
| **Cognitive and Behavioral Measures** | | |
| IQ [Mean (SD)] | 119.14 (12.04) |  |
| Mini Mental State Examination [Mean (SD)] | 28.80 (1.25) |  |
| Digit Symbol Pairing [Mean (SD)] | 13.74 (4.86) |  |
| Digit Symbol Free Recall [Mean (SD)] | 7.93 (1.10) |  |
| Hamilton Depression Rating Scale [Mean (SD)] | 3.82 (5.15) |  |
| N=number; %=percentage; SD=standard deviation; IQ Digit Symbol Pairing and Digit Symbol Free Recall were obtained in all participants with the WASI-II | |  |

**
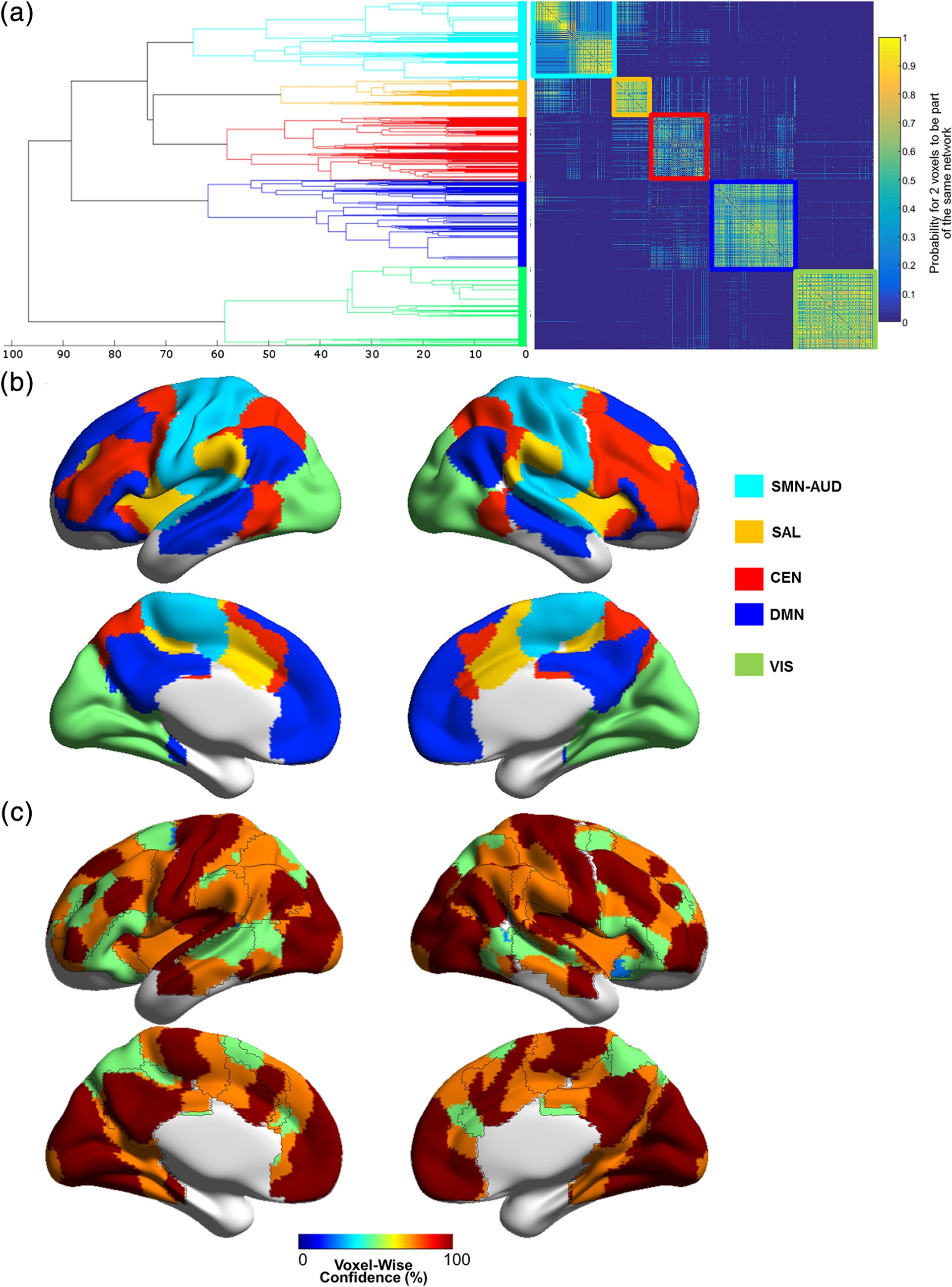
Supplementary Figure S1. Spatial distribution of the resting-state networks**

Sensorimotor Network (SMN); Salience Network (SAL); Central Executive Network (CEN);

Default Mode Network (DMN); Visual Network (VIS)

Font colors correspond to the similarly colored network maps from the Consensual Atlas of REsting‐state Networks (CAREN) [7]

1. **Supplementary Results**

**B1. Age-and-sex adjusted univariate correlations**

| **Supplementary Table S2. Age-and-sex adjusted Spearman correlation coefficients (Rho) amongst measures of fat mass, insulin-dependent glucose uptake, leptin and cortisol** | | | | | | | | |
| --- | --- | --- | --- | --- | --- | --- | --- | --- |
|  |  | **SSPG** | **FPI** | **FPG** | **BMI** | **WC** | **Leptin** | **Cortisol** |
| **SSPG** | Rho-value |  |  |  |  |  |  |  |
|  | P-value |  |  |  |  |  |  |  |
| **FPI** | Rho-value | 0.66 |  |  |  |  |  |  |
|  | P-value | 1.66E-14 |  |  |  |  |  |  |
| **FPG** | Rho-value | 0.28 | 0.33 |  |  |  |  |  |
|  | P-value | 0.004 | 0.001 |  |  |  |  |  |
| **BMI** | Rho-value | 0.57 | 0.53 | 0.25 |  |  |  |  |
|  | P-value | 1.49E-10 | 1.01E-08 | 0.01 |  |  |  |  |
| **WC** | Rho-value | 0.63 | 0.54 | 0.25 | 0.84 |  |  |  |
|  | P-value | 9.24E-13 | 2.31E-09 | 0.01 | <1E-16 |  |  |  |
| **Leptin** | Rho-value | 0.51 | 0.52 | 0.32 | 0.74 | 0.73 |  |  |
|  | P-value | 5.01E-08 | 1.31E-08 | 0.001 | <1E-16 | <1E-16 |  |  |
| **Cortisol** | Rho-value | -0.13 | -0.09 | 0.03 | -0.04 | -0.06 | -0.19 |  |
|  | P-value | 0.20 | 0.35 | 0.79 | 0.65 | 0.53 | 0.05 |  |
| BMI=body mass index; FPI=fasting plasma insulin, FPG=fasting plasma glucose; SSPG=steady-state-plasma glucose; WC=waist circumference. | | | | | | | | |

| **Supplementary Table S3. Age-and-sex adjusted Spearman correlation coefficients (Rho) amongst measures of fat mass, insulin-dependent glucose uptake, leptin and cortisol and hippocampal functional cohesiveness** | | | | | | | | |
| --- | --- | --- | --- | --- | --- | --- | --- | --- |
|  | | **SSPG** | **FPI** | **FPG** | **BMI** | **WC** | **Leptin** | **Cortisol** |
| **Left Hippocampal Cohesiveness** | Rho-value | **-0.24** | **-0.19** | **-0.15** | **-0.24** | **-0.14** | **-0.06** | **0.03** |
|  | P-value | **0.01** | **0.05** | **0.13** | **0.01** | **0.16** | **0.52** | **0.80** |
| **Right Hippocampal Cohesiveness** | Rho-value | **-0.21** | **-0.13** | **-0.19** | **-0.25** | **-0.16** | **-0.18** | **0.06** |
|  | P-value | **0.03** | **0.19** | **0.06** | **0.01** | **0.10** | **0.08** | **0.52** |
| BMI=body mass index; FPI=fasting plasma insulin, FPG=fasting plasma glucose; SSPG=steady-state-plasma glucose; WC=waist circumference. | | | | | | | | |

**B.2 Results of k-means analysis using metabolic measures as input features**

K-mean clustering identified two clusters with a majority vote of 8 fit indices (while other solutions were supported by 4 or less). Cluster 1 comprised of 44 individuals and clusters 2 comprised of 60 individuals. The clusters differed significantly in BMI [cluster 1: mean (SD)=31.57(4.03); cluster 2: mean(SD)=27.48(2.89)], in leptin [cluster 1: mean(SD)=52.81(32.39); cluster 2: mean(SD)=19.94 (13.42) and SSPG (cluster 1: mean(SD)=200.68(50.62); cluster 2: mean(SD)=101.26 (44.62)] (all P=values <0.0001). The metabolic profiles of the clusters indicated greater metabolic deviance for cluster 1 and included more females (P=0.001) but showed no other differences in cognitive and behavioral measures.

**B.3 Differences in hippocampal connectivity between metabolic k-means clusters**

As for the main analyses, clusters defined only on metabolic measures differed significantly in all measures of hippocampal functional connectivity as shown in Supplementary Figure 2.

**Supplementary Figure S2. Cluster differences in hippocampal functional cohesiveness and integration**


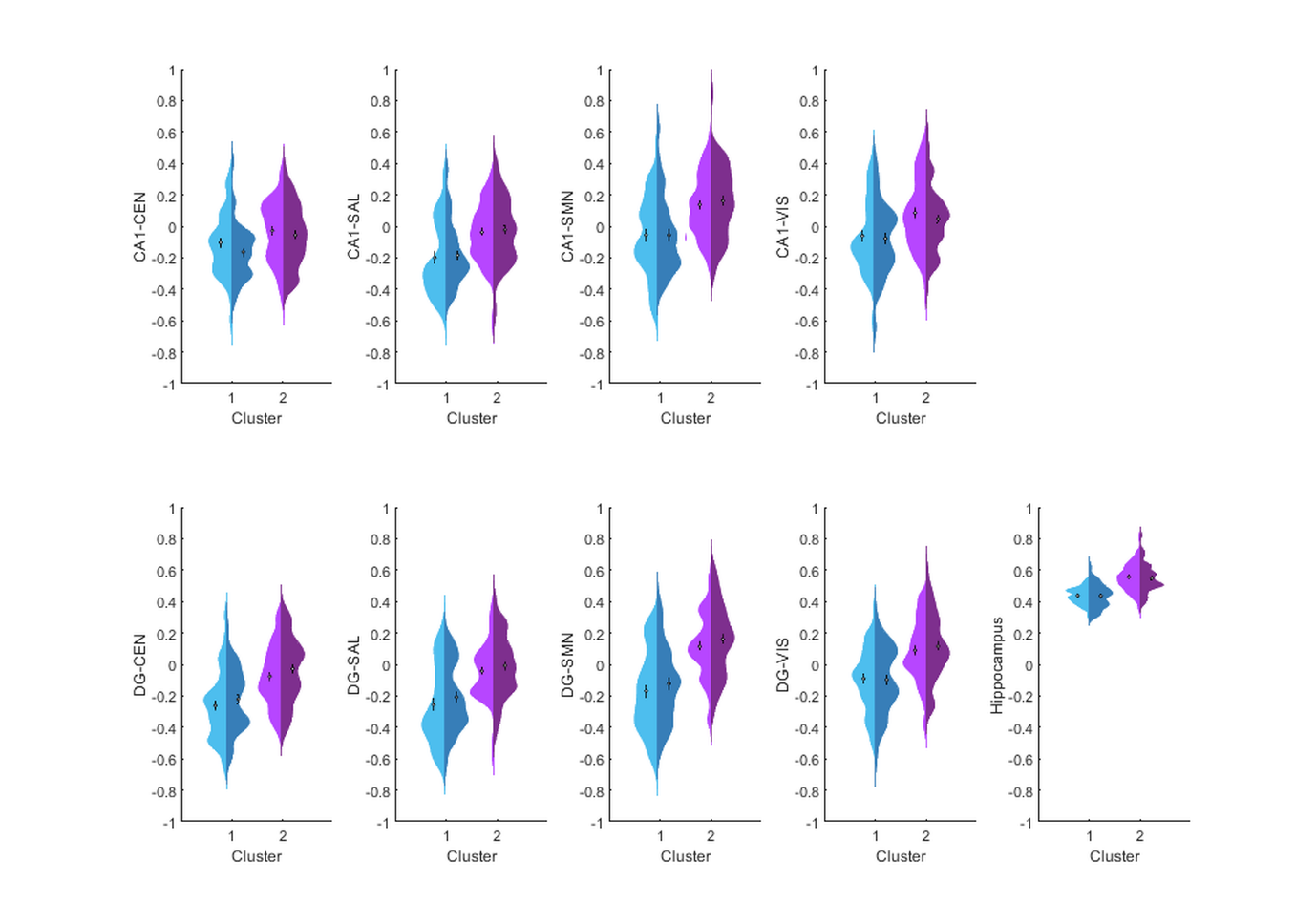


Violin plots of the distribution and differences in the hippocampal functional connectivity in clusters 1 and 2 defined only on metabolic measures; cluster 1 showed significantly lower hippocampal cohesiveness (i.e., within-hippocampus connectivity) and integration (i.e., connectivity between the left and right anterior and posterior hippocampal segment with each resting-state network).

Cluster 1 is depicted in blue and cluster 2 in purple; within each cluster the lighter hue corresponds to the left side and the darker hue to the right side. All measures of connectivity depicted are statistically significantly different between the clusters. The box plots represent the mean and standard error. CEN=central executive network; Hipp=hippocampal cohesiveness; SAL=salience network; SMN=sensorimotor network; VIS=visual network; aHipp= anterior hippocampal segment; pHipp=posterior hippocampal segment.

**References**

1. Folstein MF. The Mini-Mental State Examination. Arch Gen Psychiatry. 1983;40(7):812.

2. First, Michael B. Structured Clinical Interview for the DSM-IV Axis I Disorders: SCID-I/P, Version 2.0. New York: Biometrics Research Dept., New York State Psychiatric Institute, 1997.

3. Marcus DS, Harms MP, Snyder AZ, Jenkinson M, Wilson JA, Glasser MF, et al. Human Connectome Project informatics: quality control, database services, and data visualization. Neuroimage. 2013;80:202–19.

4. Power JD, Barnes KA, Snyder AZ, Schlaggar BL, Petersen SE. Spurious but systematic correlations in functional connectivity MRI networks arise from subject motion. Neuroimage. 2012 ;59(3):2142–54.

5. Friston KJ, Williams S, Howard R, Frackowiak RS, Turner R. Movement-related effects in fMRI time-series. Magn Reson Med. 1996;35(3):346–55.

6. Cordes D, Haughton VM, Arfanakis K, Carew JD, Turski PA, Moritz CH, et al. Frequencies contributing to functional connectivity in the cerebral cortex in “resting-state” data. AJNR Am J Neuroradiol. 2001;22(7):1326–33.

7. Doucet GE, Lee WH, Frangou S. Evaluation of the spatial variability in the major resting‐state networks across human brain functional atlases. Hum Brain Mapp. 2019;40(15):4577–87.
